# Targeting Autoimmune Myocarditis with Lemon Balm Extract: In Vivo Molecular Approach

**DOI:** 10.3390/ijms27114761

**Published:** 2026-05-25

**Authors:** Nevena Lazarevic, Marijana Andjic, Marina Nikolic, Aleksandar Kocovic, Jovana Novakovic, Jasmina Sretenovic, Vladimir Zivkovic, Vladimir Jakovljevic, Sergey Bolevich, Isidora Milosavljevic

**Affiliations:** 1 Department of Pharmacy, Faculty of Medical Sciences, University of Kragujevac, Svetozara Markovica 69, 34000 Kragujevac, Serbia; andjicmarijana10@gmail.com (M.A.); salekkg91@gmail.com (A.K.); jovana.novakovic@fmn.kg.ac.rs (J.N.); isidora.milosavljevic@fmn.kg.ac.rs (I.M.); 2 Center of Excellence for Redox Balance Research in Cardiovascular and Metabolic Disorders, Svetozara Markovica 69, 34000 Kragujevac, Serbia; marina.rankovic.95@gmail.com (M.N.); drj.sretenovic@gmail.com (J.S.); vladimirziv@gmail.com (V.Z.); drvladakgbg@yahoo.com (V.J.); 3 Department of Human Pathology, I.M. Sechenov First Moscow State Medical University, Trubetskaya Street 8, Str. 2, 119991 Moscow, Russia; bolevich2011@yandex.ru; 4 Department of Physiology, Faculty of Medical Sciences, University of Kragujevac, Svetozara Markovica 69, 34000 Kragujevac, Serbia; 5 Department of Pharmacology, I.M. Sechenov First Moscow State Medical University, Trubetskaya Street 8, Str. 2, 119435 Moscow, Russia

**Keywords:** autoimmune myocarditis, *Melissa officinalis*, lemon balm, rosmarinic acid, heart, dilative cardiomyopathy

## Abstract

Due to the complex pathophysiology and serious outcomes of autoimmune myocarditis, we sought to determine whether ethanolic lemon balm extract (LBE) could attenuate disease progression and development of dilative cardiomyopathy (DCM). EAM was induced in Dark Agouti rats by immunization with porcine myosin. Fifty animals were allocated to five groups: healthy controls, untreated EAM, and EAM treated with LBE (50, 100, or 200 mg/kg) for six weeks. Hemodynamic parameters were monitored, and echocardiography assessed cardiac structure and function. Inflammatory, oxidative, fibrotic, and apoptotic markers were analyzed. Immunological profiling revealed that LBE significantly decreased proinflammatory cytokines (IL-1, IL-6, TNF-α, IL-4, IL-17) while restoring anti-inflammatory IL-10 levels (*p* < 0.05). Antioxidant activity was confirmed by reduced levels of O_2_^−^, H_2_O_2_, and TBARS, accompanied by significant increases in SOD, CAT, and GSH activity (*p* < 0.05), and upregulation of SOD1 and SOD2 gene expression. Additionally, LBE (200 mg/kg) markedly reversed fibrotic remodeling through suppression of TGF-β expression and collagen deposition, as shown by Sirius Red staining, and mitigated apoptosis by modulating Bax/Bcl-2 balance and reducing TUNEL-positive cells. Collectively, these findings suggest that LBE exerts strong cardioprotective effects in EAM by regulating inflammatory, oxidative, fibrotic, and apoptotic pathways, thereby preventing myocarditis progression toward DCM.

## 1. Introduction

Myocarditis is an inflammatory disease of the myocardium that may arise from infectious or non-infectious causes. According to the Dallas criteria, it is defined by the presence of non-ischemic inflammatory infiltrates in myocardial tissue associated with cardiomyocyte degeneration or necrosis. The autoimmune form of myocarditis is non-infectious, and represents a consequence of autoimmune response of autoreactive T-cells on heart autoantigens (myosine, troponine), either as part of systemic diseases or as a distinct form, giant cell myocarditis [1]. Globally, about 1.8 million people develop myocarditis annually, with a higher incidence in men and younger adults [2]. The disease poses a major health burden due to its nonspecific clinical presentation, which often delays diagnosis. Consequence of acute myocarditis can be severe, since it may trigger malignant arrhythmias or cardiogenic shock, while chronic inflammation can lead to dilative cardiomyopathy (DCM), heart failure, and the need for transplantation. Notably, up to 30% of non-ischemic DCM cases are attributable to myocarditis, which is also an important cause of sudden cardiac death in young individuals [3,4]. The transition from acute to chronic myocarditis arises when the initial immune response fails to resolve, resulting in persistent inflammation and maladaptive cardiac remodeling. Sustained immune activation and cytokine release promote fibrosis, cardiomyocyte apoptosis, and ventricular dilation, ultimately contributing to the development of dilative cardiomyopathy [4].

Emerging evidence underscores the potential of phytotherapy as an innovative and safe strategy in the management of myocarditis. Bioactive plant-derived compounds or extracts particularly those rich in polyphenols and flavonoids, have demonstrated the capacity to attenuate oxidative stress, suppress pro-inflammatory mediators, and mitigate adverse myocardial remodeling. All of that suggests that phytotherapy may complement conventional treatments by targeting multiple pathogenic mechanisms underlying myocardial injury [5]. *Melissa officinalis* L. (*Lamiaceae*), otherwise called lemon balm or bee balm, is a perrenial aromatic plant, well known in traditional medicine for centuries for its beneficial effects on human health [6,7,8]. It is recognized as a valuable source of various bioactive compounds present in both its leaves and essential oil, particularly polyphenols, flavonoids, and terpenes. Numerous studies have identified several major polyphenolic acids in lemon balm extracts, occurring in different proportions. The most abundant and biologically important constituent is rosmarinic acid, considered responsible for many of the plant’s health-promoting effects [9]. Alongside rosmarinic acid, other phenolic acids have been reported, including caffeic, chlorogenic, cinnamic, gallic, protocatechuic, gentisic, p-coumaric, ferulic, salvianolic, and ellagic acids [6,10,11,12]. The therapeutic potential of lemon balm is largely attributed to its high phenolic content, due to the well-known antioxidant activity of these compounds. Besides phenolic acids, lemon balm active constituents also include flavonoids such as quercetin, quercitrin, catechin, luteolin, apigenin and volatile terpenoids [13]. Numerous data suggest strong anti-inflammatory and antioxidant potential of lemon balm extracts in different pathologies [14]. However, data on its effect autoimmune cardiovascular disease such as autoimmune myocarditis is yet to be elucidated. Our previous studies have proved cardioprotective properties of ethanolic lemon balm extract in EAM models on rats; however, molecular mechanisms of these effects have not been fully understood [15].

Thus, this study aimed to evaluate the protective and therapeutic potential of lemon balm (*Melissa officinalis* L.) extract in the context of chronic phase experimental autoimmune myocarditis with the emphasis on the prevention of dilative cardiomyopathy. Given the complex pathophysiology of autoimmune myocarditis, which involves immune cell activation, excessive production of pro-inflammatory cytokines, oxidative stress, and subsequent myocardial damage, we sought to determine whether lemon balm extract could attenuate disease progression and DCM through its known antioxidant and anti-inflammatory properties. Specifically, we investigated its effects on systemic and cardiac oxidative stress markers, redox balance, pro- and anti-inflammatory cytokine profiles, and biochemical indicators of myocardial injury. By applying an in vivo molecular approach, our goal was to provide mechanistic insight into how lemon balm extract may modulate immune-mediated myocardial inflammation and to explore its potential as a complementary therapeutic strategy for autoimmune myocarditis.

## 2. Results

### 2.1. Effect of LBE Supplementation on Hemodynamic Parameters in EAM

No significant differences between groups on day 0 (baseline values) were observed in any of the three measured parameters SBP, DBP or HR. Induction of experimental autoimmune myocarditis have led to significant drop of SBP and HR after six weeks compared to baseline values in EAM group (*p* < 0.05). On day 42, the EAM group showed significantly higher values of SBP compared to the CTRL group, while the treatment with LBE in all three doses applied restored SBP similarly to CTRL values. No significant changes in the levels of DBP were noticed between groups on day 42. After six weeks of observation, HR increased in the EAM group compared to healthy rats (*p* < 0.05); however, the treatment with LBE did not restore these values as in the control group of rats (*p* > 0.05; Table 1).

**Figure 1:**
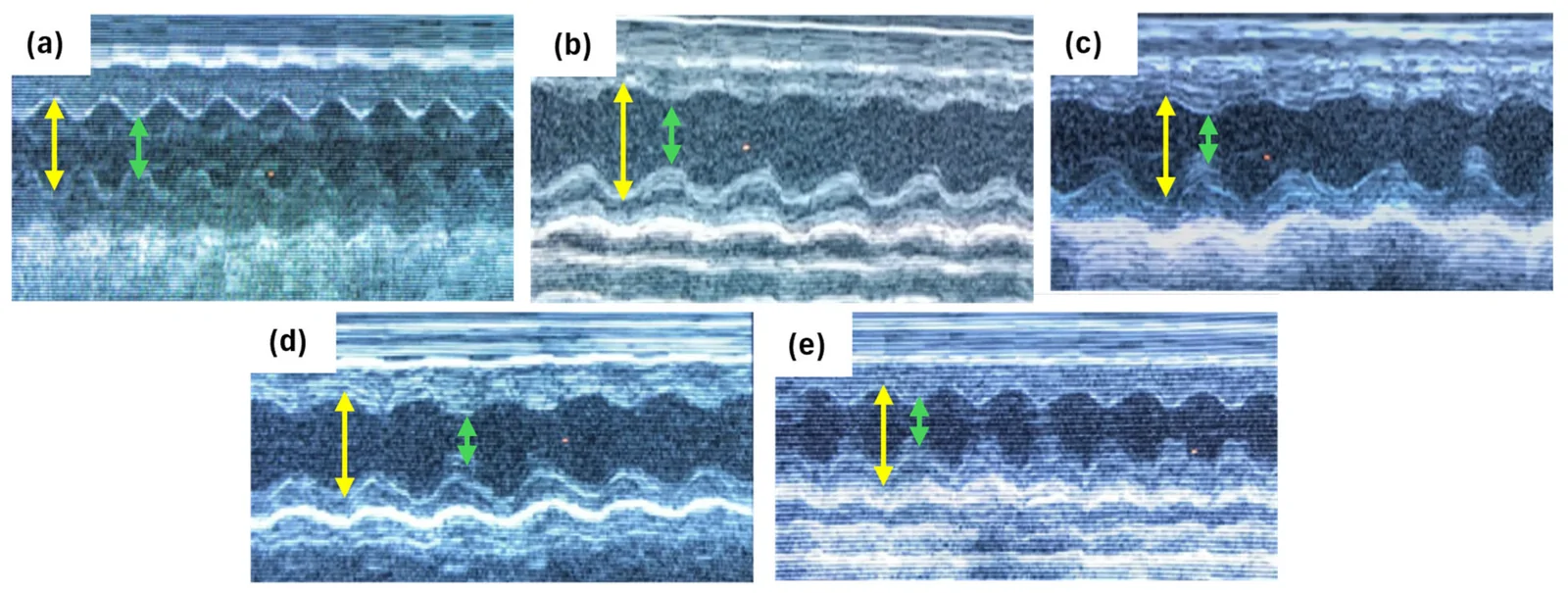
Representative M-mode echocardiograms of all experimental groups in the EAM model. Echocardiograms of rats belonging to the following groups (**a**) CTRL; (**b**) EAM; (**c**) LBE50; (**d**) LBE100; (**e**) LBE200. CTRL—healthy non-treated rats; EAM—rats with experimental autoimmune myocarditis; LBE50, LBE100 and LBE200—rats with experimental autoimmune myocarditis treated with lemon balm extract in a dose of 50 mg/kg, 100 mg/kg, or 200 mg/kg. Yellow markings indicate LVIDd (left ventricular internal diameter at end-diastole), while green markings indicate LVIDs (left ventricular internal diameter at end-systole).

Baseline values of all investigated echocardiographic parameters did not significantly differ between groups, confirming the health state of animals at the beginning of the experimental protocol. Importantly, all measured parameters differed significantly over time in all EAM groups compared to baseline values within all groups with EAM except CTRL, thus confirming the EAM model (*p* < 0.05). A significant reduction in ejection fraction (EF) was observed in the EAM group compared to the control (70.44% vs. 81.57%, *p* < 0.05). Neither of the applied doses of lemon balm extract improved EF (*p* > 0.05), and no dose-dependent differences were found. A similar trend was observed for fractional shortening (FS), which was markedly lower in the EAM group compared to CTRL (*p* < 0.05), with no improvement following LBE treatment. Both EF and FS decreased over time in all EAM groups (*p* < 0.05). Posterior wall thickness (LVPWd and LVPWs) was markedly higher in the EAM group (*p* < 0.05) in comparison with healthy hearts (CTRL). However, LBE reduced LVPWd in all three doses, but only the high-dose LBE200 significantly reduced systolic thickness (*p* < 0.05). Septal thickness (IVSd and IVSs) was significantly elevated in EAM rats (*p* < 0.05) but was unaffected by LBE treatment, regardless of dose applied. Left ventricular internal dimensions (LVIDd and LVIDs) were enlarged in the EAM group compared to CTRL, while only high-dose LBE200 significantly reduced these values (*p* < 0.05), though not to control levels (Table 2, Figure 1).

### 2.2. Effect of LBE Supplementation on Systemic Redox Status in EAM

**Figure 2:**
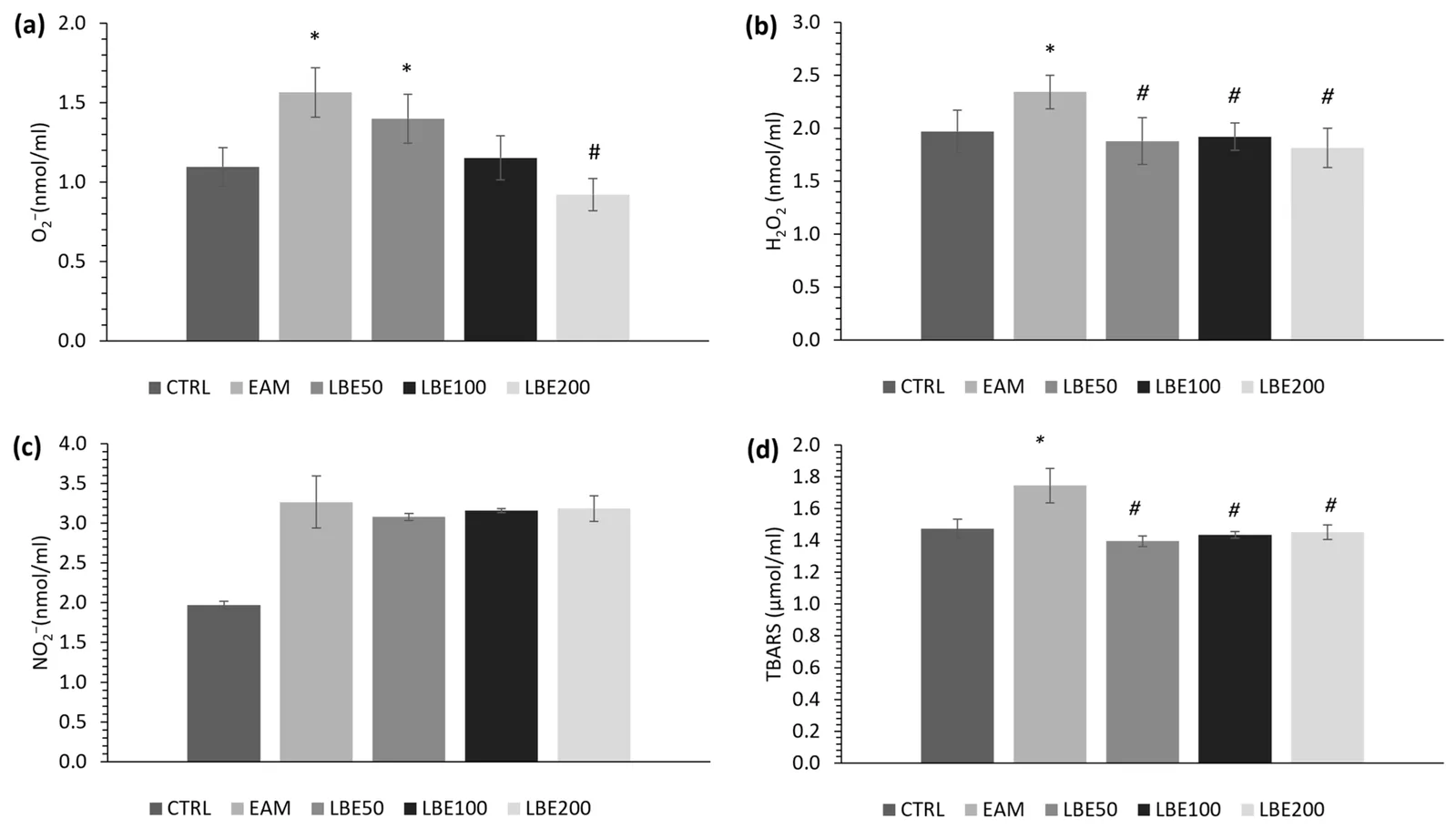
Effects of LBE on the level of prooxidants (**a**) O_2_^−^—superoxide anion radical (**b**) H_2_O_2_—hydrogen peroxide (**c**) NO_2_^−^ nitrites (**d**) TBARS—thiobarbituric acid reactive substances. CTRL—healthy non-treated rats; EAM—rats with experimental autoimmune myocarditis; LBE50, LBE100 and LBE200—rats with experimental autoimmune myocarditis treated with lemon balm extract in a dose of 50 mg/kg, 100 mg/kg or 200 mg/kg. Statistical significance at the level *p* < 0.05 compared to * CTRL group; # EAM group. Data are presented as means ± standard deviation (X ± SD).

**Figure 3:**
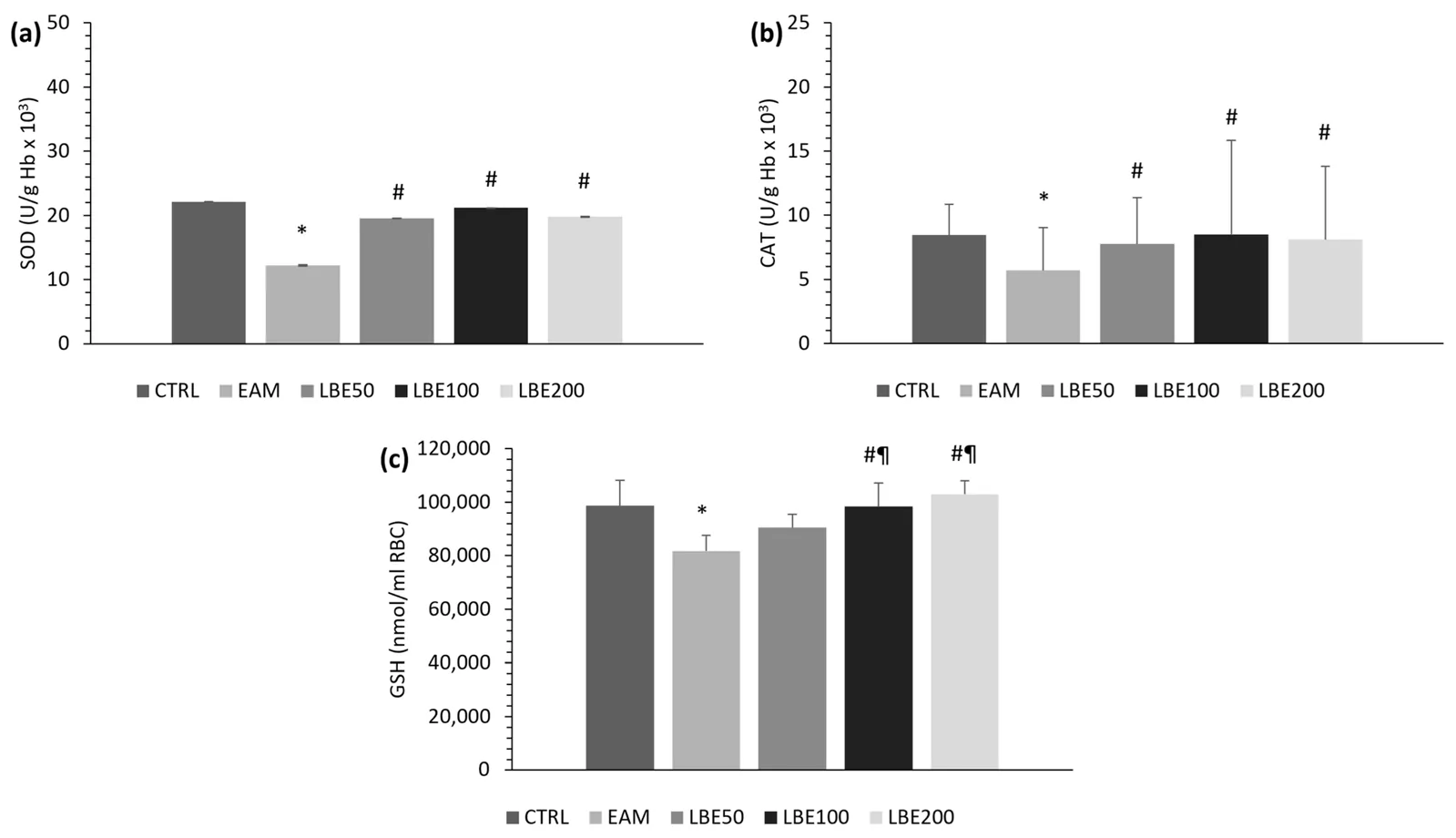
Effects of LBE on antioxidants. (**a**) SOD—superoxide dismutase (**b**) CAT—catalase (**c**) GSH—reduced glutathione. CTRL—healthy non-treated rats; EAM—rats with experimental autoimmune myocarditis; LBE50, LBE100 and LBE200—rats with experimental autoimmune myocarditis treated with lemon balm extract in a dose of 50 mg/kg, 100 mg/kg or 200 mg/kg. Statistical significance at the level *p* < 0.05 compared to * CTRL group; # EAM group; ¶ LBE50 group. Data are presented as means ± standard deviation (X ± SD).

Induction of autoimmune myocarditis significantly increased the release of all measured prooxidants (O_2_^−^, H_2_O_2_, TBARS) besides nitrites compared to healthy control rats (*p* < 0.05). However, six-week treatment with LBE succeeded in preventing the release of TBARS and H_2_O_2_ compared to EAM rats, independent of the dose applied (Figure 1; *p* < 0.05). Nonetheless, dose-dependent effect of LBE on TBARS and H_2_O_2_ was not observed (*p* > 0.05). On the other hand, only the highest dose of LBE significantly decreased the level of superoxide anion radical compared to the EAM group (*p* < 0.05), while medium and low doses were not successful in lowering O_2_^−^ compared to non-treated rats with EAM. No significant changes in the level of nitrites between groups were observed (*p* > 0.05; Figure 2). Significantly lower activity of antioxidant enzymes SOD and CAT, and the level of reduced glutathione were measured in EAM group compared to healthy animals of the CTRL group (*p* < 0.05). All three applied doses of LBE have significantly elevated the activity of these two enzymes (SOD and CAT) compared to the EAM group. However, no significant dose-dependent effect on SOD and CAT was observed between tested doses of LBE (*p* > 0.05). The level of GSH was, however, influenced only by medium and high doses of LBE (*p* < 0.05), while LBE50 did not induce any significant changes in GSH compared to EAM rats. Also, there was no significant difference in the GSH values between groups treated with medium and high doses of LBE (*p* > 0.05; Figure 3).

### 2.3. Effect of LBE Supplementation on Biochemical Markers in EAM

**Figure 4:**
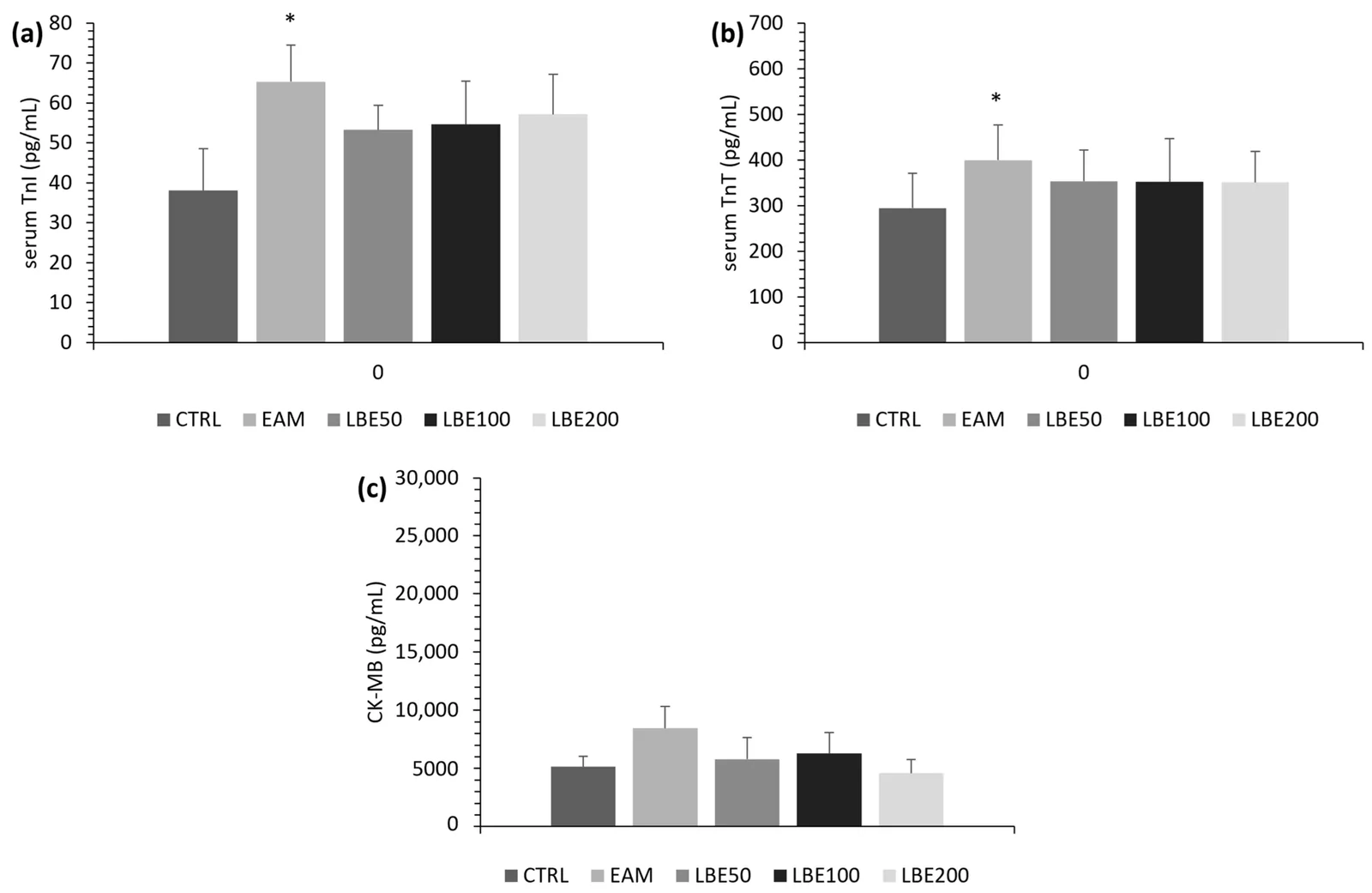
Effects of LBE on cardiac biochemical markers. (**a**) TnI—troponin I (**b**) TnT—troponin T (**c**) CK/MB—creatine kinase MB. CTRL—healthy non-treated rats; EAM—rats with experimental autoimmune myocarditis; LBE50, LBE100 and LBE200—rats with experimental autoimmune myocarditis treated with lemon balm extract in a dose of 50 mg/kg, 100 mg/kg or 200 mg/kg. Statistical significance at the level *p* < 0.05 compared to * CTRL group. Data are presented as means ± standard deviation (X ± SD).

After six weeks of observation, in the chronic phase of EAM, significant elevation of both TnI and TnT have been noticed in the EAM group compared to healthy rats. Nonetheless, LBE did not induce any changes in the measured parameters in either of applied doses (*p* > 0.05). Additionally, no significant changes in the activity of cardiac enzyme CK-MB were observed between experimental groups (Figure 4).

### 2.4. Effect of LBE Supplementation on Inflammatory Status in EAM

**Figure 5:**
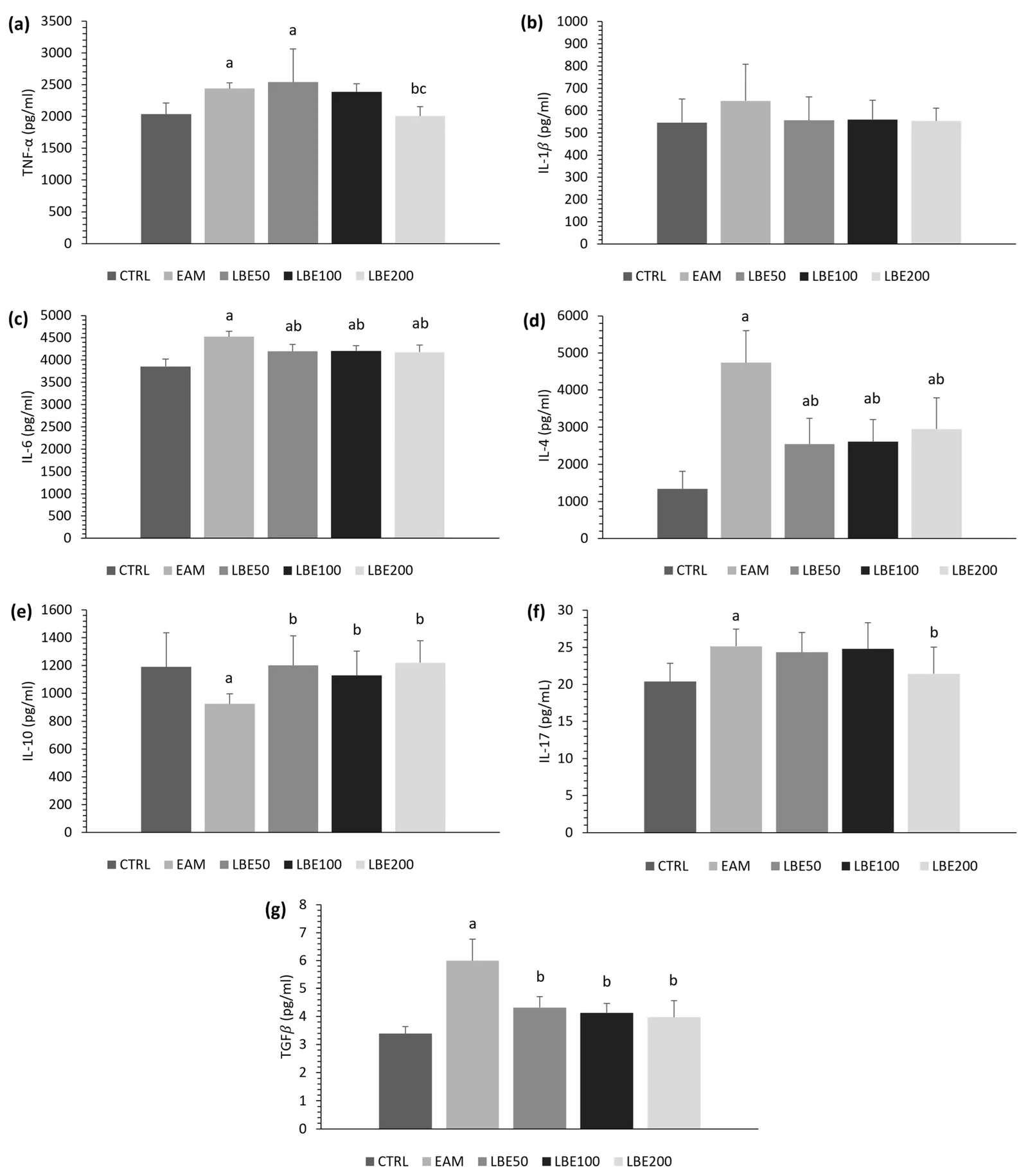
Effects of LBE on the level of cytokines. (**a**) TNF-alpha—tumor necrosis factor (**b**) IL1b–interleukin 1 beta (**c**) IL-6—interleukin 6 (**d**) IL-4—interleukin 4 (**e**) IL-10—interleukin 4 (**f**) IL17—interleukine 10 (**g**) TGFb-TGF beta. CTRL—healthy non-treated rats; EAM—rats with experimental autoimmune myocarditis; LBE50, LBE100 and LBE200—rats with experimental autoimmune myocarditis treated with lemon balm extract in a dose of 50 mg/kg, 100 mg/kg or 200 mg/kg. Statistical significance at the level *p* < 0.05 compared to ^a^ CTRL group; ^b^ EAM group; ^c^ LBE50 group. Data are presented as means ± standard deviation (X ± SD).

Induction of EAM resulted in a statistically significant increase in pro-inflammatory cytokines TNF-α, IL-6, IL-1, IL-4, IL-17, and TGF-β, accompanied by a reduction in the anti-inflammatory cytokine IL-10 (*p* < 0.05). The most pronounced anti-inflammatory response was observed following administration of the highest dose of LBE (200 mg/kg), as evidenced by a significant drop in TNF-α, IL-1β, IL-4, IL-17, and TGF-β levels compared to the EAM rats (*p* < 0.05). Although IL-6 levels were slightly elevated in the EAM group relative to other experimental groups, LBE treatment did not induce a significant reduction in IL-6 level. Notably, all three doses of LBE effectively restored IL-10 levels to values similar to the control group (Figure 5).

### 2.5. Effect of LBE Supplementation on the Expression of Genes in Myocardium in EAM

**Figure 6:**
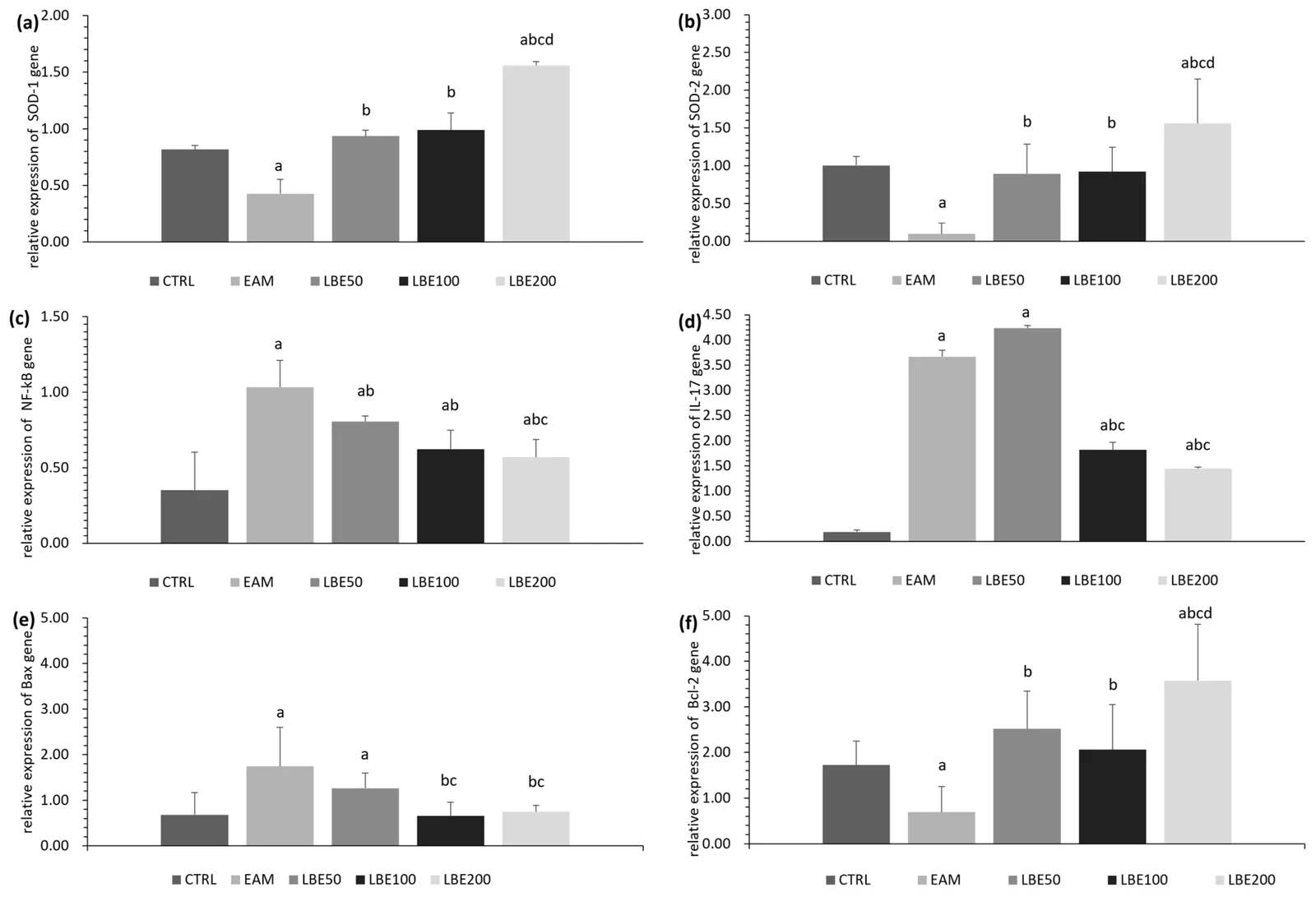
Effects of LBE on the gene expression in myocardium. (**a**) SOD-1—superoxide dismutase 1 (**b**) SOD-2—superoxide dismutase 2 (**c**) NF-kb—nuclear factor kappa b (**d**) IL17—interleukin 17 (**e**) Bax (**f**) Bcl2. CTRL—healthy non-treated rats; EAM—rats with experimental autoimmune myocarditis; LBE50, LBE100 and LBE200—rats with experimental autoimmune myocarditis treated with lemon balm extract in a dose of 50 mg/kg, 100 mg/kg or 200 mg/kg. Statistical significance at the level *p* < 0.05 compared to ^a^ CTRL group; ^b^ EAM group; ^c^ LBE50 group, ^d^ LBE50 group. Data are presented as means ± standard deviation (X ± SD).

Expression of antioxidant genes SOD1 and SOD2 was markedly lowered in the EAM group, compared to healthy control animals, while LBE in all three doses significantly upregulated the expression SOD1 and SOD2 compared to EAM. The most prominent decrease in SOD1 and SOD2 expression was achieved in the LBE200 group (*p* < 0.05; Figure 6a,b). EAM induction significantly increased myocardial proapoptotic Bax gene expression in both doses compared to healthy controls. Markedly higher nfKB and IL17 gene expression was noticed in the EAM group compared to CTRL, while supplementation with LBE in medium and high doses significantly lowered these values, especially in the LBE group relative to EAM rats. (*p* < 0.05, Figure 6c,d). However, medium and high doses of LBE markedly reduced Bax expression relative to untreated EAM and LBE50 groups (*p* < 0.05), while the low dose had no significant effect. EAM induction led to a reduction in anti-apoptotic gene Bcl-2 expression compared to controls. All LBE doses increased Bcl-2 levels compared to EAM rats, with the highest expression observed in the LBE200 group, significantly exceeding all other groups (*p* < 0.05; Figure 6e,f).

### 2.6. Histological Analysis of the Hearts in EAM (Tunnel Assay, H/E Staining, Sirius Red Staining)

**Figure 7:**
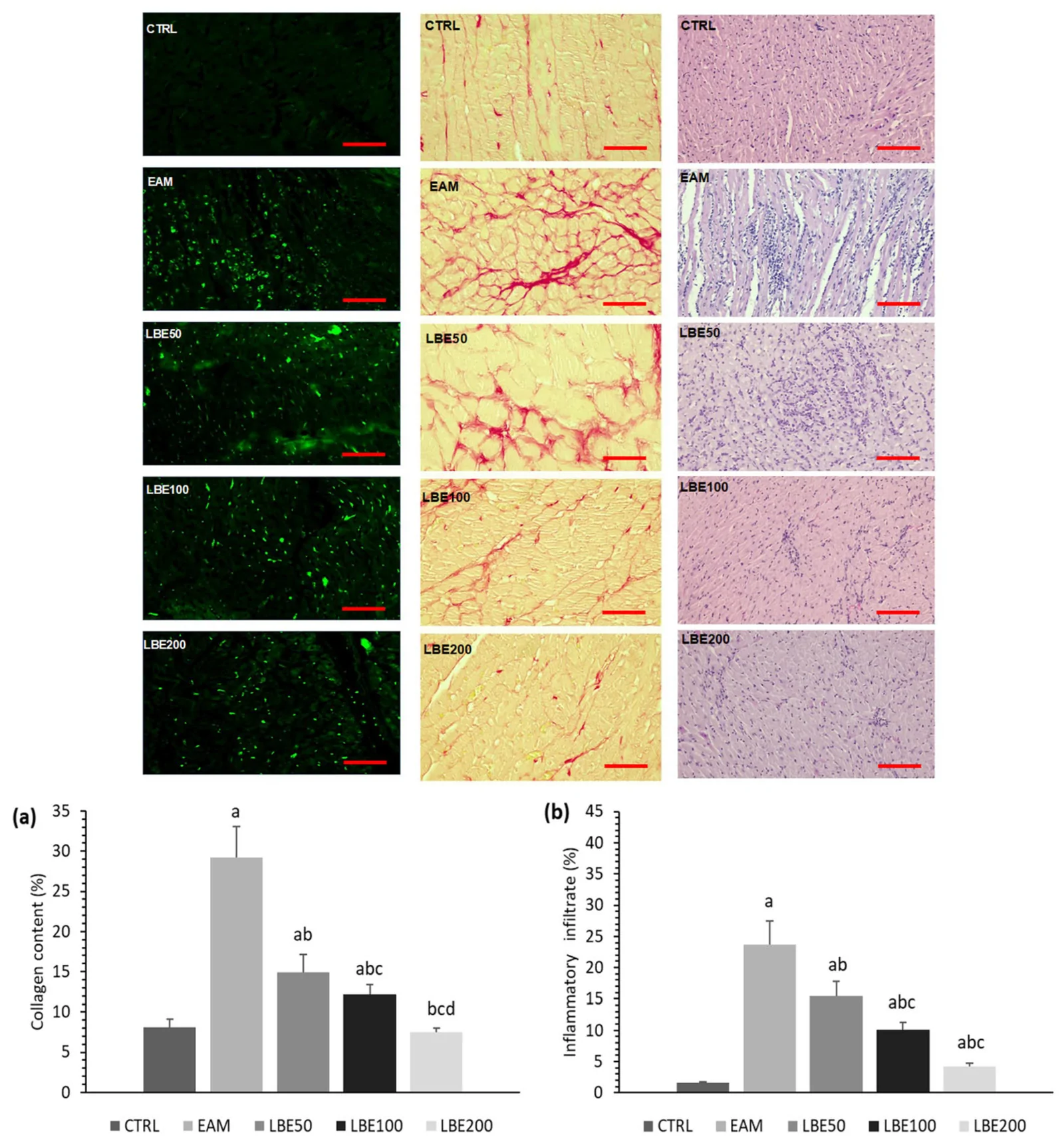
Histological analysis of the heart. tunnel assay, Sirius red stainin and hematoxylin eosin staining. (**a**) collagen content; (**b**) inflammatory infiltrate. Magnification 20× scale bar = 50 μm CTRL—healthy non-treated rats; EAM—rats with experimental autoimmune myocarditis; LBE50, LBE100 and LBE200—rats with experimental autoimmune myocarditis treated with lemon balm extract in a dose of 50 mg/kg, 100 mg/kg or 200 mg/kg. Statistical significance at the level of 0.05: a—compared to CTRL; b—compared to EAM; c—compared to LBE50; d—compared to LBE100 groups.

As expected, no fluorescent signal or TUNEL-positive cells were observed in healthy control groups. In contrast, untreated EAM animals exhibited a marked presence of TUNEL-positive cells, particularly within inflammatory infiltrates. Treatment with LBE reduced the number of apoptotic cells, most notably in the LBE200 group (Figure 7). Collagen fibers (stained red) were clearly observed in immunized, untreated EAM rats, while minimal deposition was seen in healthy controls. Treatment with all three LBE doses reduced collagen accumulation, with the most prominent effect in the LBE200 group. These visual findings were confirmed by quantitative image analysis. EAM rats exhibited significantly higher myocardial collagen content compared to controls, indicating progressive fibrosis in the chronic phase of EAM (*p* < 0.05; Figure 7a). Intact myocardial architecture without necrosis or degeneration was observed in the CTRL group of healthy animals. In contrast, the EAM group exhibited severe myocarditis with extensive inflammatory infiltration, myocardial fiber destruction, necrosis, edema, and cytoplasmic hypereosinophilia. Inflammatory infiltrates included mononuclear cells, neutrophils, and multinucleated giant cells, predominantly located in the epicardial ventricular wall. LBE treatment—especially at 200 mg/kg—substantially improved myocardial structure after 6 weeks (Figure 7). Quantitative analysis showed significantly elevated infiltration in EAM in comparison with CTRL (*p* < 0.05), while LBE treatment significantly reduced infiltration at all doses (*p* <0.05), with stronger effects at higher doses (*p* < 0.05, Figure 7b).

## 3. Discussion

Experimental autoimmune myocarditis (EAM) in rats leads to severe myocardial architectural disruption, including massive inflammatory infiltration and impaired cardiac function, often progressing to dilated cardiomyopathy (DCM) in the chronic phase. DCM is characterized by ventricular dilation and systolic dysfunction, potentially resulting in irreversible damage, fibrosis, and heart failure, with heart transplantation as the only therapeutic option [1].

The pathogenesis of EAM involves complex interactions between immune dysregulation, excessive production of proinflammatory cytokines, reactive oxygen species (ROSs), fibrosis, and apoptosis. Persistent inflammation and oxidative stress contribute to myocardial damage, while apoptotic cell death and fibrotic remodeling further impair cardiac structure and function. Therefore, in this study we sought to analyze inflammatory parameters to evaluate immune activation and modulation of Th1/Th2/Th17 responses, oxidative stress markers were assessed due to their central role in inflammation-mediated myocardial injury, and apoptotic markers because cardiomyocyte apoptosis also contributes to disease progression and cardiac dysfunction. Additionally, cardiac functional and morphological parameters were evaluated to determine whether these molecular and histopathological changes influenced cardiac performance.

Development of DCM was also confirmed in this study. Morphometric analysis revealed increased heart and spleen mass and elevated Hw/Bw and Sw/Bw ratios in untreated EAM rats six weeks post-immunization, confirming successful disease induction and enlargement of the heart. High-dose LBE treatment (200 mg/kg) for 6 weeks reduced these parameters.

Additionally, echocardiography showed a marked reduction in ejection fraction (EF) and fractional shortening (FS), along with posterior wall thickening (LVPWd, LVPWs) in EAM animals. These changes, accompanied by increased cardiomyocyte area and diameter, reflect the onset of EAM-induced DCM. Similar reductions in EF and FS have been reported in other EAM studies. Notably, the posterior wall thickening may result not only from hypertrophy but also from inflammatory infiltration and edema, as suggested by histological analysis. LBE treatment for 6 weeks, especially at medium and high doses, significantly improved EF, FS, myocardial morphology, and reduced heart mass and cardiomyocyte dimensions, thereby preventing ventricular remodeling and progression to DCM. At day 42, EAM rats showed persistent dysfunction, with continued EF and FS reduction, ventricular dilation (LVIDd, LVIDs), and wall thickening—indicative of both hypertrophy and dilation typical of chronic EAM. These findings align with previous studies showing partial EF recovery in later EAM stages due to inflammation resolution and scar formation [16,17,18,19].

Blood pressure analysis revealed significantly lower SBP in EAM animals v. control animals, in line with previous studies [20,21]. SBP values were normalized with LBE treatment in all doses, suggesting a protective effect against EAM-induced hypotension. Notably, high-dose LBE200 showed a hypotensive effect, potentially via calcium channel blockade and NO/prostacyclin pathways [22,23,24]. Clinically, reduced SBP in myocarditis is associated with poor prognosis and progression to DCM, which was also evident here—SBP correlated with EF, FS, and ventricle dilation. No significant LBE-induced HR change was observed at day 42, although HR naturally declined in chronic EAM, likely reflecting impaired cardiac function due to fibrosis and remodeling. Elevated levels of cardiac troponin T (TnT) in EAM rats on day 21 post-immunization have been previously reported [25,26], while increased troponin I (TnI) levels at the same time point have also been confirmed in mice [27], corresponding to the peak of myocardial inflammation and injury. Nearly three decades ago, researchers studied the kinetics of cardiac injury markers CK-MB and TnT in the EAM mouse model and found that their circulating levels change over the disease course. Specifically, the highest sensitivity of these markers was observed between days 16 and 21 post-immunization, coinciding with maximal myocardial necrosis and inflammation. However, after day 23, levels start to decline due to the replacement of inflammation by fibrosis in the chronic phase (day 42). Moreover, TnT was suggested to be a more sensitive marker of inflammatory myocardial injury than CK-MB [28]. Our results are consistent with these findings. Six-week LBE treatment did not significantly affect serum levels of CK-MB, TnT, or TnI at any dose. Although no prior studies have investigated the effects of LBE in inflammatory cardiac diseases, a 14-day LBE treatment at 50 and 100 mg/kg has been shown to reduce circulating TnI levels in rats subjected to LAD-induced ischemia/reperfusion injury. The cardioprotective effect was attributed to the extract’s strong antioxidant and anti-inflammatory properties [29].

It is well known that the balance between Th1, Th2, and Th17 cytokine production significantly influences the outcome of myocarditis. In the acute phase, Th1 cells and pro-inflammatory cytokines (IL-1, IL-6, TNF-α) dominate, coinciding with peak inflammation. In the chronic or recovery phase, Th2 cytokines (IL-4, IL-10, IL-13) prevail, often accompanied by fibrotic remodeling. In our study, circulating levels of IL-6 and TNF-α were elevated even after six weeks, with a moderate increase in IL-1, which may reflect sustained Th1-mediated activation and the additional role of IL-1 and IL-6 in promoting IL-17 production by T cells. Prior research has shown increased Th17 cell populations and elevated IL-17, IL-6, and IL-23 in human myocarditis and DCM, along with reduced Treg function [30]. Upregulated myocardial gene expression of these cytokines in the EAM model has also been widely reported [18,31,32], with some studies indicating a correlation between cytokine levels and disease severity [20,33]. Chronic elevation of TNF-α is also associated with progressive left ventricular remodeling, hypertrophy, dysfunction, and cardiomyopathy [34]. Our findings are consistent with this claim, as TNF-α levels remained high during chronic EAM, coinciding with histological and hemodynamic signs of myocardial dysfunction and DCM. Additionally, chronic DCM in EAM was accompanied by increased apoptosis, as indicated by elevated *Bax* expression. TNF-α is known to induce cardiomyocyte apoptosis via complex mechanisms, including TNF receptor-mediated activation of MAPK and NF-κB signaling [35]. The observed upregulation of NF-κB in EAM/6 myocardium supports this pathway, linking TNF-α-driven apoptosis and maladaptive myocardial remodeling in chronic EAM.

However, only high doses of LBE reduced plasma TNF-α levels, suggesting that its anti-apoptotic effects are primarily mediated through antioxidant mechanisms (e.g., reduced ROS production), rather than TNF-α suppression. NF-κB, a key transcription factor in the myocardium, regulates genes involved in inflammation, immunity, and cell survival. Chronic activation of NF-κB contributes to myocardial damage, inflammation, and apoptosis through increased TNF-α production. Studies have shown that p50-deficient mice are resistant to TNF-α–induced cardiomyopathy, and chronic NF-κB inhibition reduces cytokine production, apoptosis, and heart failure mortality [35]. Our findings support this, showing increased NF-κB expression in EAM rats` myocardium, correlating with elevated TNF-α and *Bax*, and suggesting chronic inflammation and remodeling. LBE reduced NF-κB expression in a dose-dependent manner, most effectively in the LBE200 group. Regulatory T cells (Tregs), crucial for immune homeostasis in EAM, act partly via IL-10 and TGF-β secretion [1,4]. In our study, IL-10 levels were significantly reduced in EAM, which is consistent with previous research. IL-10 is thought to play a more prominent role in the chronic phase, as proved in this study by a drop of IL10 in EAM rats, while LBE in restored IL-10 levels at all doses. Chronic EAM was also characterized by increased TGF-β, which LBE reduced alongside an IL-10 increase, indicating LBE’s immunomodulatory potential via Treg activation and fibrosis suppression [36]. IL-4, a pro-inflammatory Th2 cytokine involved in eosinophil recruitment, was elevated in EAM. LBE treatment markedly reduced the levels of IL-4 in all groups, regardless of dose. These results highlight LBE’s ability to modulate Th2 responses, potentially via components like rosmarinic acid and quercetin, which have shown similar effects in allergy models [37,38]. The role of Th17 cells and IL-17 in EAM progression to DCM is well-established. IL-17 levels were elevated in the EAM group, matching increased myocardial IL-17 gene expression. IL-17 promotes fibrosis via fibroblast activation and contributes to cardiac dysfunction [39]. Our results show a correlation between IL-17 and myocardial *Bax* expression, indicating pro-apoptotic effects. Nonetheless, high dose of LBE successfully reduced IL-17 levels in the chronic EAM. Additionally, IL-17 also activates NF-κB, impairing cardiomyocyte calcium homeostasis by suppressing SERCA2a and L-type Ca^2+^ channel gene expression [40]. In contrast, IL-17A knockout mice show improved contractility [41]. LBE’s cardioprotective effects may be partly due to suppression of IL-17–mediated NF-κB activation, reducing inflammation, remodeling, and DCM progression. These findings underscore LBE’s potential to modulate key immune pathways—Th1, Th2, and Th17—by reducing NF-κB activation, cytokine production, apoptosis, and fibrosis, ultimately preserving myocardial structure and function in EAM.

Cardiomyocyte death in EAM occurs via both necrosis and apoptosis, as confirmed in our study by the highest TUNEL-positive cell count and degree of necrosis observed in the untreated EAM rats. Apoptosis was more pronounced in the acute EAM rats, with LBE reducing TUNEL-positive cells in a dose-dependent manner. Correspondingly, LBE reversed EAM-induced upregulation of *Bax* and downregulation of *Bcl-2*, indicating anti-apoptotic effects via inhibition of the intrinsic pathway. These findings align with reports identifying apoptosis as a key mechanism of cardiomyocyte loss in chronic EAM and DCM. Importantly, apoptosis in EAM is not limited to cardiomyocytes but extends to myocardial fibroblasts, contributing to tissue remodeling and scar formation in chronic DCM. The anti-apoptotic effects of LBE may be partially explained by its ability to suppress pro-inflammatory cytokines such as IL-1 and TNF-α, which are known triggers of caspase activation. Additionally, LBE’s antioxidant activity—demonstrated by reduced ROS production (e.g., O_2_^−^, H_2_O_2_, NO, TBARS)—may contribute to apoptosis inhibition, as ROS are potent inducers of both intrinsic and extrinsic apoptotic pathways through mitochondrial membrane permeabilization and *Bax* activation [42]. Although limited data exist on the effects of *Melissa officinalis* extract in cardiovascular and autoimmune diseases, its anti-apoptotic properties have been demonstrated in doxorubicin-induced cardiotoxicity models, where it downregulated *Bax* and caspase-3 expression at higher doses than those used in our study [6]. These actions are largely attributed to rosmarinic acid, the primary bioactive compound, which has been shown to reduce apoptosis in various pathologies by downregulating *Bax*, caspase-3, and increasing *Bcl-2* [43].

In our study, EAM induction was histopathologically confirmed by marked inflammatory infiltration and cardiac fibrosis consistent with previous EAM rat model studies. EAM hearts were characterized with inflammation and predominantly fibrosis, as expected in the chronic phase of EAM evaluated in this study, as evidenced by increased inflammatory infiltration and increased collagen content in EAM rats. Echocardiographic findings and elevated TGF-β further supported the development of DCM in the chronic phase of EAM. Six-week LBE administration improved myocardial architecture and also decreased collagen deposition and TGF-β levels in all doses, indicating anti-fibrotic potential. This is supported by previous studies in other cardiac injury models, where LBE and its constituents reduced fibrosis via modulation of profibrotic mediators such as TGF-β, angiotensin II, and endothelin-1 [6,43,44,45]. The anti-fibrotic effects of LBE may stem from the synergistic action of its bioactive compounds—particularly rosmarinic acid, oleanolic and ursolic acids, and flavonoids like quercetin, rutin, catechin, and epigallocatechin—all of which are known to reduce myocardial fibrosis in EAM or other models through suppression of cytokines (IL-6, IL-17A, TNF-α), inhibition of fibroblast proliferation, or ECM deposition [43,44,45,46]. LBE also significantly reduced inflammatory infiltration in EAM, likely due to its strong anti-inflammatory properties, supported by reduced levels of IL-1, IL-4, IL-6, IL-17, and TNF-α. These effects may be attributed to LBE’s phenolic and flavonoid components. Rosmarinic acid, the primary compound, has shown potent anti-inflammatory effects across autoimmune models by downregulating COX-2 and inflammatory cytokines. It also modulates NF-κB and activates PPAR-γ, particularly in ischemia/reperfusion injury models [43]. Several studies have suggested that some plant extracts exert cardioprotective effects in EAM and other type of inflammatory cardiovascular diseases through antioxidant and anti-inflammatory mechanisms similar to those observed in our study. For example, natural compounds such quercetin, apigenin, luteolin and green tea polyphenols were shown to attenuate oxidative stress, suppress pro-inflammatory cytokine production, and reduce myocardial fibrosis and remodeling in the EAM model [8]. Additionally, lemon balm has already been investigated in clinical studies involving anxiety, stress-related disorders, metabolic disturbances, and mild cardiovascular dysfunction, where it demonstrated a favorable safety profile, as it is classified as GRAS (generally considered safe) together with antioxidant and anti-inflammatory properties [8]. Since it is easy to cultivate at relatively low production costs, and has established uses in traditional medicine, this plant represents a promising candidate for scalable phytopharmaceutical development. Furthermore, considering its multitarget anti-inflammatory, antioxidant, antifibrotic, and antiapoptotic effects, LBE could potentially be explored as an adjunctive complementary therapy alongside conventional treatment strategies in myocarditis. However, further pharmacokinetic investigations, extract standardization, and controlled clinical trials are required before clinical translation can be achieved.

## 4. Materials and Methods

### 4.1. Plant Material and Extract Preparation

For the purposes of this study, dried leaves of *Melissa officinalis* L. (Lamiaceae) were used. The plant material—dried lemon balm leaves (*Melissae folium*)—was purchased from the company Bilje Borča d.o.o. (Belgrade, Serbia, Lot: 030-12-19). The dried material was ground into a fine powder using a laboratory mill (IKA A11, IKA^®^ Werke GmbH & Co., Staufen im Breisgau, Germany) and stored in well-sealed paper bags at room temperature until the extraction process began.

Lemon balm extract (LBE) was prepared using the reflux extraction method with 70% ethanol as the solvent. The powdered plant material (*M. officinalis* L. leaves) was mixed with a 10-fold volume of 70% ethanol and subjected to heating in a reflux apparatus equipped with a water-cooled condenser. The extraction was carried out at the boiling point of the solvent for 2.5 h. Following extraction, the mixture was filtered through gauze to remove coarse plant residues. The obtained liquid extract was then left at room temperature to allow spontaneous sedimentation of ballast substances. The supernatant was further purified by filtration through Whatman No. 1 filter paper (Cytiva, Little Chalfont, Buckinghamshire, UK). The obtained purified extract was then concentrated to dryness using a rotary vacuum evaporator (RV05 basic IKA, IKA^®^ Werke GmbH & Co., Staufen im Breisgau, Germany) at 40 °C, a flow rate of 90 g/min, and under 250 mbar vacuum pressure. The resulting dry extract was collected and stored in dark glass bottles at +4 °C until further analysis. Upon completion, the dry extract yield was quantified [47]. Chemical characterization of the used extract is available in our previous paper [14].

### 4.2. Ethical Concerns

All animal experiments conducted in this study were reviewed and approved by the Ethics Committee for the Welfare of Experimental Animals at the Faculty of Medical Sciences, University of Kragujevac (Kragujevac, Serbia), under approval number 01-10171, approval date 4 November 2020. The procedures adhered to the guidelines set forth in the European Directive 2010/63/EU on the protection of animals used for scientific purposes, complied with the principles of Good Laboratory Practice (GLP), and followed the provisions of the earlier Directive 86/609/EEC concerning the care and use of laboratory animals.

### 4.3. Animals

A total of 50 male *Dark Agouti* rats (8 weeks old, body weight 160 ± 20 g) were included in the study. The animals were obtained from the vivarium of the Military Medical Academy in Belgrade, Serbia, a for an accredited facility for laboratory animal breeding. Prior to the start of the experiment, all animals underwent a 14-day quarantine period for health assessment and acclimatization. They were housed in polyethylene cages (four rats per cage) under standardized laboratory conditions (22 ± 2 °C temperature, 12 h light/dark cycle). Rats had free access (*ad libitum*) to water and a standard laboratory diet composed of 9% fat, 20% protein, and 53% carbohydrates. Animals were monitored daily for general health status, behavior, and signs of distress throughout the experimental period.

### 4.4. Study Protocol

All animals (*n* = 50) were randomly divided into five groups using identification numbers and a random number generator to ensure equal group size (10 per group): CTRL—healthy, non-treated animals; EAM—animals with induced experimental autoimmune myocarditis; LBE50, LBE100, LBE200—animals with induced experimental autoimmune myocarditis treated with LBE in a dose of either 50 mg/kg, 100 mg/kg or 200 mg/kg. LBE was dissolved in tap water, each day, prior to *per os* treatment of animals. Animals received LBE once a day, every day at the same time (volume 400 µL) for six weeks, i.e., 42 days.

### 4.5. Induction of Disease

Purified porcine cardiac myosin (Sigma Aldrich, Munich, Germany), enriched with *Mycobacterium tuberculosis* H37Ra (Difco Laboratories, Detroit, MI, USA), was used as the antigen for EAM induction. Initially, the purified cardiac myosin was dissolved in phosphate-buffered saline (PBS), while an equal volume of Complete Freund’s Adjuvant (CFA, Sigma Aldrich), supplemented with *M. tuberculosis* (10 mg/mL), was prepared separately. The contents of both tubes were then combined and vortexed. The resulting suspension was homogenized using two syringes connected via a Luer-Lock adapter by repeatedly pushing the contents back and forth for 60 min. The final emulsion was drawn into a sterile 1 mL syringe with a Luer-Lock tip and a 26G needle. The emulsion was prepared *ex tempore*, on the day of immunization. On day 0 of the experimental protocol, rats were injected subcutaneously with 0.1 mL of the final emulsion into the footpads of both hind paws (0.05 mL per paw), corresponding to an immunizing dose of 0.25 mg of cardiac myosin per rat. The control group received an emulsion of CFA and PBS without cardiac myosin [48].

### 4.6. Blood Pressure and Heart Rate Measurement

Baseline systolic and diastolic blood pressure (SBP, DBP; mmHg) and heart rate (HR; bpm) were measured in all animals prior to the induction of experimental autoimmune myocarditis. Measurements were performed using a non-invasive tail-cuff plethysmography system (Rat Tail Cuff Method Blood Pressure System, MRBP-R, IITC Life Science Inc., Los Angeles, CA, USA), which detects vascular pressure via a sensor placed on the tail. Each animal underwent 8–10 consecutive measurements, and the mean value was used for analysis. Assessments were conducted at two time points: on day 0 (baseline) to confirm group homogeneity, and at the end of the protocol—on day 42, prior to euthanasia [16].

### 4.7. Transthoracic Echocardiography

To assess the effects of LBE on in vivo cardiac function and the progression of autoimmune myocarditis, all animals underwent transthoracic echocardiographic evaluation. The procedure was performed twice—on day 0 (baseline), to verify health status and group homogeneity, and at the end of the experimental protocol (day 42), immediately prior to sacrifice. Animals were anesthetized via intraperitoneal injection of ketamine (75 mg/kg) and xylazine (5 mg/kg). Echocardiographic imaging was performed using a Hewlett-Packard Sonos 5500 ultrasound system (Andover, MA, USA), equipped with a 15.0 MHz transducer. In the long-axis parasternal view, M-mode recordings were obtained by positioning the cursor perpendicular to the interventricular septum and the posterior wall of the left ventricle (LV) at the level of the papillary muscles. The following parameters were measured in M-mode and expressed in millimeters (mm):

- Interventricular septal thickness at end-diastole (IVSd);
- Left ventricular internal diameter at end-diastole (LVIDd);
- Posterior wall thickness of the LV at end-diastole (LVPWd);
- Interventricular septal thickness at end-systole (IVSs);
- Left ventricular internal diameter at end-systole (LVIDs);
- Posterior wall thickness of the LV at end-systole (LVPWs).

Fractional shortening (FS%) was calculated using the M-mode measurements according to the following equation:

$$FS(\%)=\frac{\left(LVIDd-LVIDs\right)}{LVIDd}\;\times \;100$$

where LVIDd represents the left ventricular internal diameter at end-diastole, and LVIDs denotes the internal diameter at end-systole.

Ejection fraction (EF%) was calculated using the Teichholz formula, where LVEDV and LVESV represent left ventricular end-diastolic and end-systolic volumes, respectively:

$$EF\left(\%\right)=\frac{LVEDV-LVESV}{LVEDV}\;\times \;100\;LVEDV=\frac{7\times LVIDd}{2.4\times LVIDd}\;LVESV=\frac{7\times LVIDs}{2.4\times LVIDs}$$

All calculations were performed based on M-mode measurements obtained from the parasternal long-axis view [49,50].

### 4.8. Biochemical Analyses

Upon completion of the experimental protocol, all animals were anesthetized with ketamine (5 mg/kg) and xylazine (75 mg/kg) and sacrificed by decapitation. Blood samples were collected, and serum was separated using a gel-based method for subsequent biochemical and immunological analyses. Serum concentrations of cardiac biomarkers, including creatine phosphokinase MB isoform (CK-MB FineTest, Wuhan, China, Cat. ER0841), cardiac troponin T (cTnT, FineTest, Wuhan, China; Cat. ER1396), cardiac troponine I ELISA kit (FineTest, Wuhan, China; CatR0870H096) and C-reactive protein (CRP, FineTest, Wuhan, China, Cat. ER1048), were quantified by high-sensitivity enzyme-linked immunosorbent assay (ELISA) according to manufacturer`s instructions.

### 4.9. Oxidative Stress Analyses

Blood samples were collected from the jugular vein at sacrifice. Plasma and erythrocytes were separated by centrifugation for subsequent analyses oxidative stress parameters spectrophotometrically. In plasma, pro-oxidant markers were determined, including thiobarbituric acid-reactive substances (TBARSs), superoxide anion radical (O_2_^−^), hydrogen peroxide (H_2_O_2_), and nitrites (NO_2_^−^). In erythrocyte lysates, antioxidant status was assessed through measurement of reduced glutathione (GSH) and the enzymatic activities of superoxide dismutase (SOD) and catalase (CAT). Lipid peroxidation (TBARS) was quantified spectrophotometrically at 530 nm, nitrites by the Griess reaction (550 nm), superoxide anion using nitro blue tetrazolium (NBT, 530 nm), and hydrogen peroxide through the phenol red method catalyzed by peroxidase (610 nm). Antioxidant parameters were determined as follows: GSH by reaction with DTNB (412 nm), CAT activity by monitoring H_2_O_2_ decomposition at 360 nm, and SOD activity using the epinephrine autoxidation method (470 nm) [51].

### 4.10. Evaluation of Proinflammatory and Antiinflammatory Markers

Furthermore, levels of pro- and anti-inflammatory cytokines—tumor necrosis factor-alpha (TNF-α), interleukins 1, 6, 4, 10, and 17 (IL-1, IL-6, IL-4, IL-10, IL-17), and transforming growth factor-beta (TGF-β)—were determined. All measurements were performed using the sandwich ELISA technique with commercially available kits validated for rat biological samples.

### 4.11. Histological Processing and Cardiac Tissue Analysis

Following isolation, each heart was weighed and transversely sectioned to separate the left and right halves for subsequent histological processing. The tissues were fixed in 4% paraformaldehyde (neutral-buffered), then dehydrated in graded ethanol solutions (70%, 96%, and 100%), cleared with xylene, embedded in paraffin, and sectioned at a thickness of 5 μm. For morphological assessment and collagen visualization, sections were stained with hematoxylin and eosin (H&E) and Picrosirius red, respectively. Tissue imaging was conducted using an Olympus BX51 light microscope (Olympus Corporation, Tokyo, Japan) The extent of cellular infiltration and collagen deposition was analyzed using Image Pro-Plus software 7.0 (Media Cybernetics, Rockville, MD, USA). To ensure representative sampling, 10 non-consecutive sections were analyzed per heart, taken at 100 μm intervals (i.e., every 20th section). The degree of infiltration and collagen content was quantified and expressed as a percentage. In the control group, no inflammatory infiltrate was observed, and this parameter was accordingly recorded as 0%. Collagen quantification and evaluation of the extent of cellular infiltration was performed independently by two researchers, and the final value was calculated as the average of their measurements.

### 4.12. Immunohistochemical Analysis—TUNEL Assay

To evaluate the degree of apoptosis in myocardial tissue affected by experimental autoimmune myocarditis (EAM), as well as the potential antiapoptotic effects of chronic lemon balm extract (LBE) administration, the TUNEL assay (Terminal deoxynucleotidyl transferase dUTP Nick-End Labeling) was performed. Cardiac tissues from all experimental and control groups were subjected to this analysis. The TUNEL method is based on the enzymatic activity of endonucleases activated during cardiomyocyte apoptosis, which cleave phosphodiester bonds in DNA, creating exposed 3′-OH terminus. These ends allow the incorporation of biotinylated, fluorescently labeled deoxyuridine triphosphates (dUTPs) by the enzyme terminal deoxynucleotidyl transferase (TdT). Labeled DNA fragments are then visualized using fluorescence microscopy. Myocardial tissue sections (5 μm thick) were stained and analyzed according to the manufacturer’s protocol for the commercial TUNEL kit (One-step TUNEL In Situ Apoptosis Kit, Elabscience, Cat. No. E-CK-A320/E-CK-A321, Houston, TX, USA).

### 4.13. Gene Expression Analyses

Relative gene expression of markers involved in apoptosis (Bcl-2, Bax), oxidative stress (SOD-1/Cu,Zn-SOD and SOD-2/Mn-SOD), and inflammation (IL-17A, NF-κB) were determined by quantitative real-time PCR (RT-qPCR), using specific commercially available primers (Table 3). Myocardial tissue samples designated for PCR analysis were stored at −80 °C until processing. Total RNA was extracted from the left ventricle using the Extractme Total RNA Kit (Blirt S.A., Gdansk, Poland; Cat. No. EM09.2, EM11.2), according to the manufacturer’s instructions. Complementary DNA (cDNA) was synthesized from 1 μg of isolated total RNA using the iScript™ Reverse Transcription Supermix for RT-qPCR (Bio-Rad Laboratories, Inc., Hercules, CA, USA; Cat. No. 1708841), following the manufacturer’s protocol.

RT-qPCR was performed using the SsoAdvanced Universal SYBR Green Supermix (Bio-Rad Laboratories, Inc., Hercules, California, USA; Cat. No. 172-5270) on the CFX96 Touch Real-Time PCR Detection System (Bio-Rad, Hercules, CA, USA). Specific primers for SOD-1, SOD-2, Bax, Bcl-2, NF-κB, and IL-17A (Invitrogen, Carlsbad, CA, USA) were used. β-actin served as the housekeeping gene.

Gene expression levels were analyzed using the 2^−ΔΔCt^ method, calculated as follows:

ΔCt = C(tgene of interest) − Ct (housekeeping gene)

ΔΔCt = ΔCt(EAM sample) − ΔCt(CTRL sample)

2^−ΔΔCt^ relative gene expression of gene of interest in EAM compared to CTRL.

Each sample was analyzed in duplicate, and the mean Ct value was used for calculation. Relative mRNA expression was expressed as the ratio of target gene expression to β-actin [51,52].

### 4.14. Statistical Analysis

Statistical analysis was performed using IBM SPSS Statistics software, version 22.0 for Windows. Sample size for the study was determined by using G-Power software, v3.1 (http://www.gpower.hhu.de/en.html, accessed on 21 May 2026). The normality of data distribution was assessed using the Kolmogorov–Smirnov and Shapiro–Wilk tests, along with visual inspection through histograms and normal Q–Q plots. To identify statistically significant differences between groups, one-way or two-way analysis of variance (One-Way ANOVA; Two-Way ANOVA) was applied, followed by Bonferroni or Tukey post hoc tests for pairwise comparisons. When assumptions for parametric testing were not met, the non-parametric Kruskal–Wallis test was used as an alternative. Data are presented as mean (M) ± standard deviation (SD), and a *p*-value of <0.05 was considered statistically significant. Graphs and tables were created using Microsoft Excel for Mac, version 2016.

## 5. Conclusions

Although this is amongst the first studies to investigate LBE potential in chronic autoimmune myocarditis, the findings strongly support its potential in modulating inflammation and fibrosis, likely through the combined effects of its active constituents. In the chronic phase of experimental autoimmune myocarditis (EAM), six-week treatment with LBE attenuated pathological myocardial remodeling and prevented progression toward dilated cardiomyopathy by reducing left ventricular wall thickening and myocardial hypertrophy. Histological analysis demonstrated marked attenuation of myocardial fibrosis and collagen deposition, accompanied by reduced inflammatory infiltrate in a dose-dependent manner, with the highest dose showing the most pronounced cardioprotective effects. Six-week LBE treatment improved myocardial redox balance by reducing oxidative stress and increasing antioxidant defense, including upregulation of antioxidant gene expression. It also modulated chronic inflammatory signaling through suppression of systemic proinflammatory cytokines, particularly the Th17-associated response, as evidenced by decreased systemic IL-17 levels and reduced myocardial expression of IL-17 and NF-κB genes. Furthermore, LBE exerted significant antiapoptotic effects by regulating Bax/Bcl-2 expression and decreasing apoptotic cell death in myocardial tissue. Further studies are needed to define the specific inflammatory cell populations affected by LBE treatment in EAM. In conclusion, the cardioprotective effects of LBE observed in this study are likely mediated through inhibition of apoptosis via antioxidant, anti-inflammatory, and gene-regulatory mechanisms, though further research is required to elucidate its precise molecular targets, especially within the extrinsic apoptotic pathway.

## Figures and Tables

**Table 1:** Effects of LBE on systolic blood pressure (SBP), diastolic blood pressure (DBP) and heart rate (HR).

Group	Timepoint	SBP (mmHg)	DBP (mmHg)	HR (Beats/min)
CTRL	Day 0	124.50 ± 4.95	68.33 ± 6.66	317.50 ± 19.09
	Day 42	123.67 ± 9.11	66.67 ± 8.73	324.5 ± 7.77
EAM	Day 0	128.00 ± 7.52	70.55 ± 15.55	296.00 ± 16.97
	Day 42	106.00 ± 3.53 *^a^	75.00 ± 11.31	375.00 ± 43.21 *^a^
LBE50	Day 0	126.00 ± 5.67	75.25 ± 6.67	320.5 ± 21.33
	Day 42	125.17 ± 8.67 ^b^	77.50 ± 6.42	369.83 ± 30.67 *
LBE100	Day 0	125.00 ± 9.78	76.80 ± 7.89	315.25 ± 20.67
	Day 42	127.00 ± 8.94 ^b^	79.67 ± 8.52	361.00 ± 33.59 *
LBE200	Day 0	129.50 ± 7.86	76.45 ± 8.23	313.75 ± 24.12
	Day 42	128.00 ± 8.53 ^b^	78.50 ± 7.82	373.5 ± 22.45 *

CTRL—healthy non-treated rats; EAM—rats with experimental autoimmune myocarditis; LBE50, LBE100 and LBE200—rats with experimental autoimmune myocarditis treated with lemon balm extract in a dose of 50 mg/kg, 100 mg/kg or 200 mg/kg. Statistical significance at the level *p* < 0.05 compared to a—CTRL group; b—EAM group; * within the group day 0 vs. day 42. Data are presented as means ± standard deviation (X ± SD).

**Table 2:** Effects of LBE on the dimensions of the left ventricle.

	**Timepoint**	**CTRL**	**EAM**	**LBE50**	**LBE100**	**LBE200**
IVSd (cm)	Day 0	0.130 ± 0.018	0.132 ± 0.001	0.118 ± 0.006	0.138 ± 0.008	0.136 ± 0.019
	Day 42	0.126 ± 0.003	0.168 ± 0.008 *^a^	0.159 ± 0.021 *^a^	0.157 ± 0.009 ^a^	0.152 ± 0.014 ^a^
LVIDd (cm)	Day 0	0.461 ± 0.039	0.479 ± 0.043	0.478 ± 0.043	0.432 ± 0.074	0.451 ± 0.074
	Day 42	0.501 ± 0.041	0.602 ± 0.014 *^a^	0.580 ± 0.055 *^a^	0.613 ± 0.047 *^a^	0.574 ± 0.079 *^ab^
LVPWd (cm)	Day 0	0.129 ± 0.009	0.147 ± 0.015	0.117 ± 0.001	0.129 ± 0.012	0.116 ± 0.004
	Day 42	0.148 ± 0.024	0.172 ± 0.022 *^a^	0.155 ± 0.017 *^b^	0.158 ± 0.048 ^b^*	0.160 ± 0.032 *^b^
IVSs (cm)	Day 0	0.125 ± 0.028	0.141 ± 0.009	0.114 ± 0.011	0.116 ± 0.012	0.108 ± 0.07
	Day 42	0.129 ± 0.032	0.203 ± 0.013 *^a^	0.189 ± 0.029 *^a^	0.207 ± 0.038 *^a^	0.178 ± 0.027 *^a^
LVIDs (cm)	Day 0	0.252 ± 0.046	0.239 ± 0.023	0.193 ± 0.038	0.201 ± 0.028	0.202 ± 0.026
	Day 42	0.277 ± 0.042	0.390 ± 0.024 *^a^	0.369 ± 0.043 *	0.385 ± 0.036 *	0.355 ± 0.068 *^b^
LVPWs (cm)	Day 0	0.131 ± 0.011	0.167 ± 0.009	0.150 ± 0.033	0.130 ± 0.017	0.140 ± 0.036
	Day 42	0.140 ± 0.014	0.212 ± 0.029 *^a^	0.199 ± 0.018 ^a^*	0.194 ± 0.033 *^a^	0.185 ± 0.026 *^ab^
FS (%)	Day 0	44.957 ± 10.932	50.050 ± 3.747	51.850 ± 9.290	53.350 ± 2.385	54.825 ± 39.95
	Day 42	44.817 ± 4.235	35.237 ± 3.056 *^a^	36.467 ± 3.717 *	37.067 ± 3.911 *	39.95 ± 5.125 *
EF (%)	Day 0	80.393 ± 10.962	86.214 ± 2.774	87.112 ± 7.606	88.863 ± 1.668	89.705 ± 3.325
	Day 42	81.569 ± 4.037	70.744 ± 3.818 *^a^	72.491 ± 4.649 *	72.840 ± 4.638 *	76.120 ± 5.808 *

IVSs, IVSd—interventricular septum endsistolic or enddiastolic (cm); LVIDd, LVIDs—left ventricular internal diameter at end systole or end diastole (cm); LVPWd, LVPWs—left ventricular posterior wall thickness at end systole or end diastole; FS—fractional shortening (%); EF—ejection fraction (%). CTRL—healthy non-treated rats; EAM—rats with experimental autoimmune myocarditis; LBE50, LBE100 and LBE200—rats with experimental autoimmune myocarditis treated with lemon balm extract in a dose of 50 mg/kg, 100 mg/kg or 200 mg/kg. Statistical significance at the level *p* < 0.05 compared to a—CTRL group; b—EAM group; * within the group day 0 vs. day 42. Data are presented as means ± standard deviation (X ± SD).

**Table 3:** Commercially available primers used for RT-qPCR analysis.

Target Gene	Forward Primer (5′-3′)	Reverse Primer (5′-3′)
β-actin	GATCAGCAAGCAGGAGTACGAT	GTAACAGTCCGCCTAGAAGCAT
Bax	GCTACAGGGTTTCATCCAGGAT	ATGTTGTTGTCCAGTTCATCGC
Bcl-2	GCAAAGCACATCCAATAAAAGCG	GTACTTCATCACGATCTCCCGG
SOD-1	TGAAGAGAGGCATGTTGGAGAC	CACACGATCTTCAATGGACACA
SOD-2	AATCAACAGACCCAAGCTAGGC	CACAATGTCACTCCTCTCCGAA
IL-17A	GCAAGAGATCCTGGTCCTGAAG	AGGTCTCTGTTTAGGACGCATG
NF-κB	GTTTGGTTTGAGACATCCCTGC	CTGTCTTATGGCTGAGGTCTGG

## Data Availability

The original contributions presented in this study are included in the article. Further inquiries can be directed to the corresponding author.

## Abbreviations

The following abbreviations are used in this manuscript:

Bax	Bcl-2-associated X protein
Bcl-2	B-cell lymphoma 2
CAT	Catalase
CFA	Complete Freund’s adjuvant
CK-MB	Creatine kinase MB isoenzyme
cDNA	Complementary DNA
CRP	C-reactive protein
CTRL	Control group
DBP	Diastolic blood pressure
DCM	Dilated cardiomyopathy
DTNB	5,5′-Dithiobis-(2-nitrobenzoic acid)
EAM	Experimental autoimmune myocarditis
EF	Ejection fraction
ELISA	Enzyme-linked immunosorbent assay
FS	Fractional shortening
GLP	Good Laboratory Practice
GSH	Reduced glutathione
H_2_O_2_	Hydrogen peroxide
HR	Heart rate
IL	Interleukin
IL-1β	Interleukin-1 beta
IL-4	Interleukin-4
IL-6	Interleukin-6
IL-10	Interleukin-10
IL-17	Interleukin-17
IVSd	Interventricular septal thickness at end-diastole
IVSs	Interventricular septal thickness at end-systole
LBE	Lemon balm extract
LVIDd	Left ventricular internal diameter at end-diastole
LVIDs	Left ventricular internal diameter at end-systole
LVPWd	Left ventricular posterior wall thickness at end-diastole
LVPWs	Left ventricular posterior wall thickness at end-systole
NBT	Nitro blue tetrazolium
NF-κB	Nuclear factor kappa B
NO_2_^−^	Nitrites
O_2_•^−^	Superoxide anion radical
PBS	Phosphate-buffered saline
RNA	Ribonucleic acid
RT-qPCR	Real-time quantitative polymerase chain reaction
SBP	Systolic blood pressure
SOD	Superoxide dismutase
TBARS	Thiobarbituric acid reactive substances
TGF-β	Transforming growth factor beta
TNF-α	Transforming growth factor beta
TnI	Troponin I
TnT	Troponin T
TUNEL	Terminal deoxynucleotidyl transferase dUTP nick-end labeling
